# Supplementary figures and images for: HIV-1 5’-Leader Mutations in Plasma Viruses Before and After the Development of Reverse Transcriptase Inhibitor-Resistance Mutations
Source: medRxiv. 2023 Aug 29:2023.06.04.23290942. Originally published 2023 Jun 5. Preprint. [Version 2] doi: 10.1101/2023.06.04.23290942 (PMC10274971; doi:10.1101/2023.06.04.23290942)

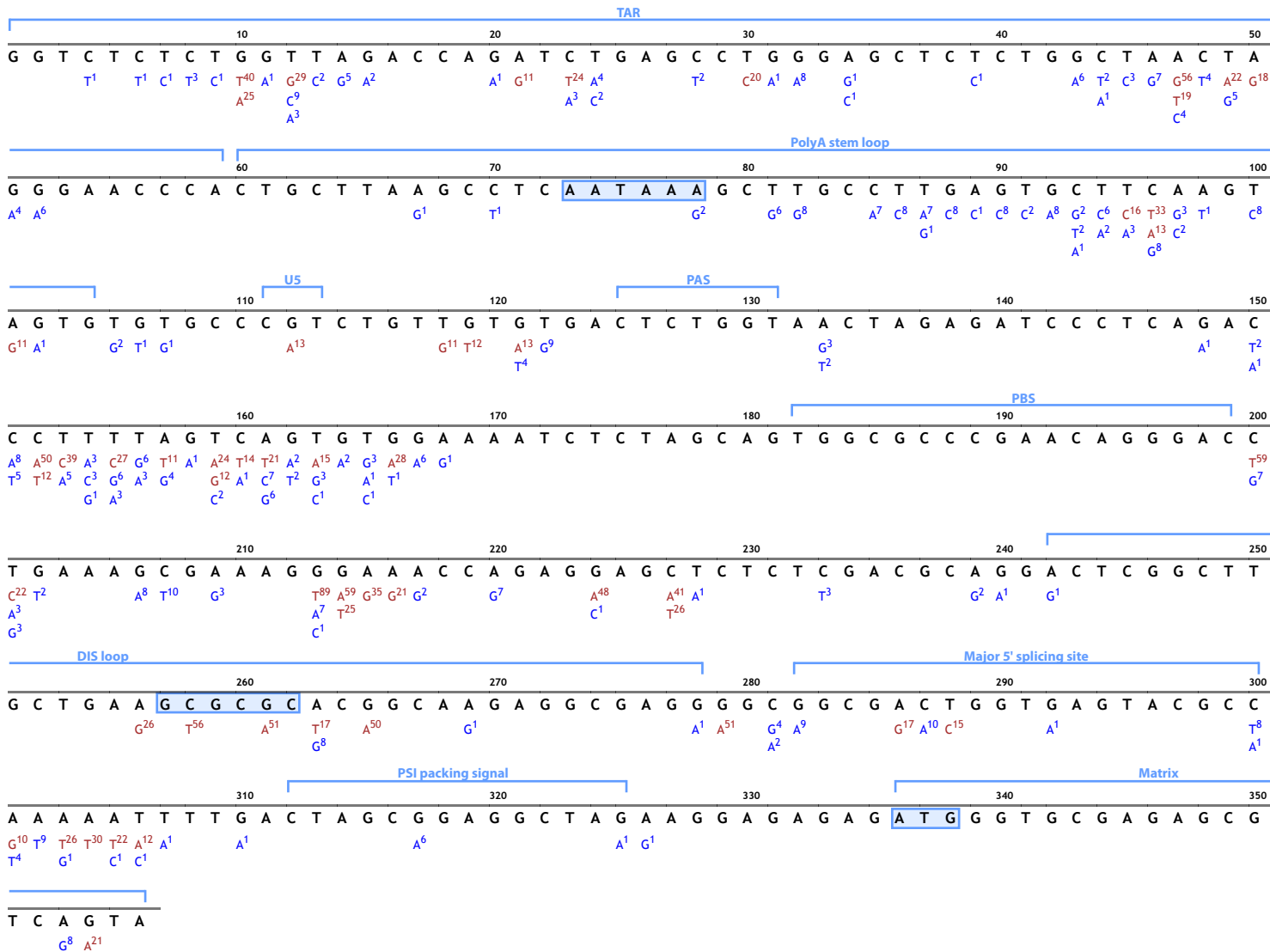

Supplement: Supplement 1 — Supplementary Figure 1 HIV-1 5-leader nucleotide differences from the HXB2 sequence observed in 1,417 sequences in the Los Alamos National Laboratories HIV Sequence Database dataset. Superscripts indicate the percentage of times that a nucleotide was reported. Highlighted regions include (1) TAR: trans-activation response element; (2) PolyA: polyadenylation signal loop with a box surrounding the poly A motif; (3) U5; (4) PAS: primer activation signal; (5) PBS: primer binding site; (6) DIS: dimer initiation signal loop with a box surrounding the palindromic DIS; (7) major 5’ splicing site; (8) PSI packaging signal; (9) Matrix protein with a box surrounding the start codon. [file media-1.pdf]

% differences from HXB2

NAs only

Indels

Naive (n=56)

Treated (n=24)

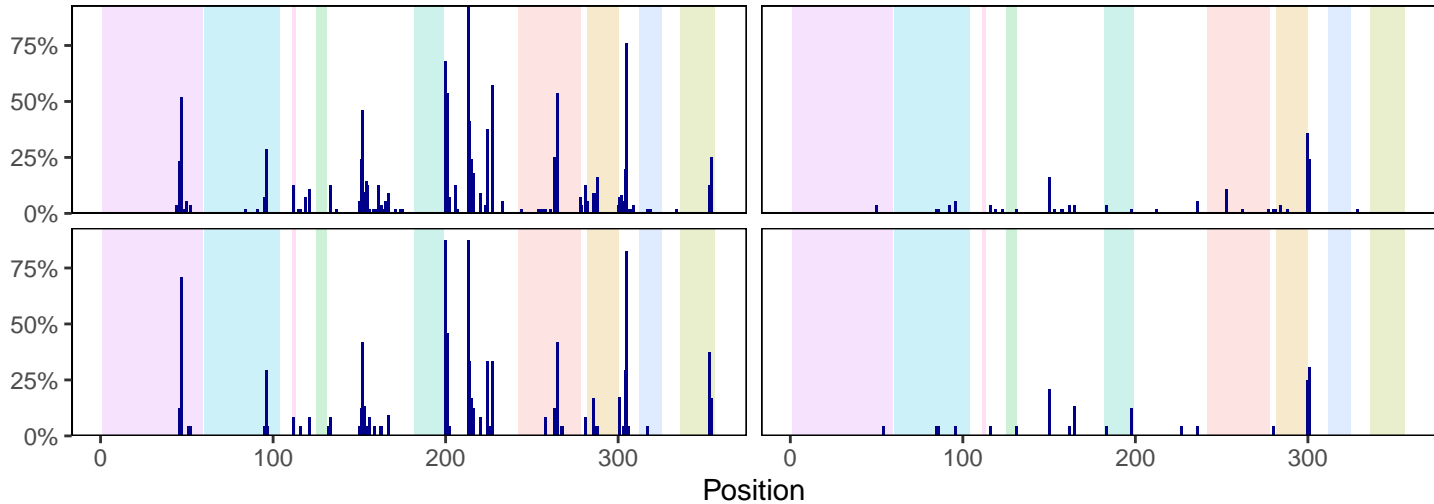

Supplement: Supplement 2 — Supplementary Figure 2 Distribution of HIV-1 5’-leader nucleotide differences from HXB2 and indels in the 56 baseline sequences from ART-naïve individuals compared with the 24 baseline sequences from ART-experienced individuals. [file media-2.pdf]
